# The Human-Bacterial Pathogen Protein Interaction Networks of *Bacillus anthracis*, *Francisella tularensis*, and *Yersinia pestis*

**DOI:** 10.1371/journal.pone.0012089

**Published:** 2010-08-09

**Authors:** Matthew D. Dyer, Chris Neff, Max Dufford, Corban G. Rivera, Donna Shattuck, Josep Bassaganya-Riera, T. M. Murali, Bruno W. Sobral

**Affiliations:** 1 Virginia Bioinformatics Institute, Blacksburg, Virginia, United States of America; 2 Myriad Genetics, Salt Lake City, Utah, United States of America; 3 Department of Computer Science, Virginia Polytechnic Institute and State University, Blacksburg, Virginia, United States of America; BMSI-A*STAR, Singapore

## Abstract

**Background:**

*Bacillus anthracis*, *Francisella tularensis*, and *Yersinia pestis* are bacterial pathogens that can cause anthrax, lethal acute pneumonic disease, and bubonic plague, respectively, and are listed as NIAID Category A priority pathogens for possible use as biological weapons. However, the interactions between human proteins and proteins in these bacteria remain poorly characterized leading to an incomplete understanding of their pathogenesis and mechanisms of immune evasion.

**Methodology:**

In this study, we used a high-throughput yeast two-hybrid assay to identify physical interactions between human proteins and proteins from each of these three pathogens. From more than 250,000 screens performed, we identified 3,073 human-*B. anthracis*, 1,383 human-*F. tularensis*, and 4,059 human-*Y. pestis* protein-protein interactions including interactions involving 304 *B. anthracis*, 52 *F. tularensis*, and 330 *Y. pestis* proteins that are uncharacterized. Computational analysis revealed that pathogen proteins preferentially interact with human proteins that are hubs and bottlenecks in the human PPI network. In addition, we computed modules of human-pathogen PPIs that are conserved amongst the three networks. Functionally, such conserved modules reveal commonalities between how the different pathogens interact with crucial host pathways involved in inflammation and immunity.

**Significance:**

These data constitute the first extensive protein interaction networks constructed for bacterial pathogens and their human hosts. This study provides novel insights into host-pathogen interactions.

## Introduction

*Bacillus anthracis*, *Francisella tularensis* and *Yersinia pestis* are known to cause pathogenesis, in part, by evading or suppressing immune responses. For instance, it is well recognized that anthrax lethal toxin (LT) is a key player in the *B. anthracis* pathogenic mechanism that induces macrophage apoptosis [1] and cleavage of MAPK at specific recognition sites [2]. *Y. pestis* suppresses local inflammation and impairs macrophage phagocytic activity through a complex type III secretion system (T3SS) and its associated protein LcrV [3]. *F. tularensis* either fails to induce an immune response or causes immune suppression by inducing transforming growth factor (TGF-β) [4]. Both *Y. pestis* and *F. tularensis* are Gram-negative bacteria that synthesize lipopolysaccharide (LPS) with poor Toll-like receptor 4 (TLR4)-stimulating activity, although *F. tularensis* can signal via TLR2 [5]. Thus, all three pathogens share similar mechanisms of pathogenesis that involve modulation of immune responses. Traditional microbiology and immunology approaches have characterized only a few pathogenic proteins for each microbe, resulting in a limited understanding of pathogenicity and evasion mechanisms.

**Figure 1:**
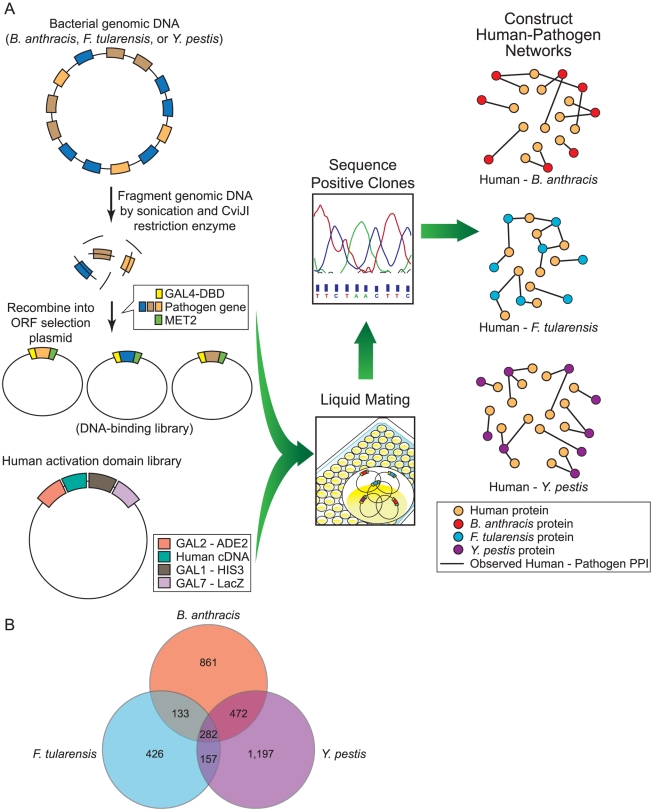
Overview of experimental workflow. A) Overview of analysis pipeline used in this study. B) Venn diagram displaying the number of human proteins interacting with each of the three pathogens in this study.

In contrast to investigating either the host or the pathogen, focusing on interactions between host and pathogen proteins may uncover hidden associations that have not been detected by traditional methods. To uncover host-pathogen protein interactions on a genome-wide scale for these three immune-evading systems and to define a target set of proteins for understanding mechanisms of pathogenicity, we designed a high-throughput yeast two-hybrid assay aimed at characterizing protein-protein interactions (PPIs) between human and bacterial proteins. We generated DNA-binding domain libraries for each pathogen and activation domain libraries containing human proteins in a haploid Matα strain of *Saccharomyces cerevisiae*. We tested for activation of the two-hybrid reporter genes using a similar protocol that was previously used for identifying interactions between proteins in *Plasmodium falciparum* [6]. We then sequenced positive colonies to identify interacting partners (see Figure 1A). In total, we performed more than 250,000 screens across the three pathogens. We obtained 3,073 PPIs between 1,748 human proteins and 943 *B. anthracis* proteins, 1,383 PPIs between 999 human proteins and 349 *F. tularensis* proteins, and 4,059 PPIs between 2,108 human proteins and 1,218 *Y. pestis* proteins. We used an independent computational analysis to study the network properties (degree and centrality) of the human proteins that interact with pathogen proteins in our dataset. Additionally, we used a graph-alignment algorithm to identify conserved subsets of human-pathogen PPIs found across multiple networks.

These data constitute the first extensive protein interaction networks constructed for bacterial pathogens and their human hosts. Typically, data detailing host-pathogen interactions is ascertained from small-scaled experiments that are designed to target specific proteins, complexes, or pathways of interest. This is evident from the number of interactions between host and bacterial pathogens currently available in seven public resources [7], [8], [9], [10], [11], [12], [13]. For example, these databases only contain one human-*B. anthracis* interaction, no human-*F. tularensis* interactions, and seven human-*Y. pestis* interactions.

## Results and Discussion

In total we identified 3,911, 1,942, and 5,157 PPIs for the human-*B. anthracis*, human-*F. tularensis*, and human-*Y. pestis* networks respectively. We filtered this set of PPIs by removing human proteins that interact with large number of pathogen proteins identified by multiple screens with other pathogens (unpublished data), reasoning that such interactions are likely to be false positives. This step yielded a final set of 3,073, 1,383, and 4,059 PPIs for the human-*B. anthracis*, human-*F. tularensis*, and human-*Y. pestis* networks respectively (see Table 1). We found that 888 human-*B. anthracis*, 167 human-*F. tularensis*, and 2,205 human-*Y. pestis* PPIs contain pathogen proteins that are labeled as “putative”, “hypothetical”, or “uncharacterized”. See Figure 1B for a comparison of the sets of human proteins found to interact with each of these pathogens.

**Table 1:** Summary of human-pathogen interactions.

Organsim	# PPIs	# PPIs^*^	# *H. sapiens*	# pathogen	# pathogen
			proteins	proteins	proteins^*^
*B. anthracis*	3,073	888	1,748	943	285
*F. tularensis*	1,383	167	999	349	66
*Y. pestis*	4,059	2,025	2,108	1,218	630

Counts in columns marked with an “*” correspond to pathogen proteins labeled as “putative”, “uncharacterized”, or “hypothetical”.

### Bacterial pathogens have evolved to interact with human hubs and bottlenecks

**Figure 2:**
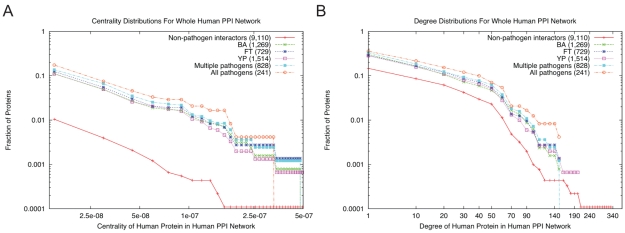
Network properties of interacting proteins. Cumulative log-log plots of (A) node centralities and (B) degrees for six subsets of nodes in the whole human protein-protein interaction network: the red curve is for the set of proteins in the human PPI network that do not interact with any pathogen in our dataset; the green line is for the set interacting with *B. anthracis*; the dark blue line is the for set interacting with *F. tularensis*; the purple line is for the set interacting with *Y. pestis*; the light blue line is for the set interacting with at least two pathogens; and the orange line is for the set interacting with all three pathogens. The fraction of proteins at a particular value of degree or centrality is the number of proteins having that value or greater divided by the number of proteins in the set. (Counts in parentheses represent the number of proteins in each set.)

Several recent studies [14], [15] have suggested that viral proteins have evolved to preferentially interact with protein hubs (proteins with many interacting partners) and bottlenecks (proteins that lie in shortest paths between many pairs of proteins) in the human PPI network. We hypothesized that bacterial proteins interact with human proteins with high degree and centrality, since pathogens may have evolved to control and disrupt essential complexes and pathways governing the host response. Our analysis supports this hypothesis. More specifically, Figure 2(a) displays a log-log plot of the degree distributions of six sets of proteins in a human PPI network collated from multiple databases [7], [8], [9], [10], [11], [12], [13]. These plots show that across almost the entire range of degrees, human proteins interacting with bacterial pathogens tend to have higher degree than proteins that do not interact with any bacterium. The betweenness centrality results display the same trend (see Figure 2(b)). We used Gene Set Enrichment Analysis (GSEA) [16] to test whether the gaps we observe in Figure 2 between the curve for all non-pathogen interactors and the other curves are statistically significant. GSEA yields *p*-values less than 10^−6^ for both degree and centrality for all sets, supporting the conclusions we draw from Figure 2. To address the possibility that the observed patterns may be artifacts of experimental biases or errors in the human PPI network, we followed an earlier approach for viral-human PPIs [15]: we repeated the GSEA analyses using two subsets of the human PPI network: (i) interactions detected by small-scale experiments and (ii) interactions observed by large-scale studies. We obtained statistically-significant results in both cases (see Table 2).

**Table 2:** GSEA results.

				Degree			Centrality	
Network	Group	# proteins	ES	# proteins	*p*-value	ES	# proteins	*p*-value
		in group		contributing			contributing	
	*B. anthracis*	1,269	0.28	834	<10^−6^	0.46	1,269	<10^−6^
	*F. tularensis*	729	0.28	574	<10^−6^	0.45	729	<10^−6^
W	*Y. pestis*	1,514	0.28	1,325	<10^−6^	0.47	1,514	<10^−6^
	Multiple	828	0.31	579	<10^−6^	0.46	828	<10^−6^
	All	241	0.32	187	<10^−6^	0.45	241	<10^−6^
	*B. anthracis*	608	0.39	608	<10^−6^	0.60	608	<10^−6^
	*F. tularensis*	373	0.38	373	10^−6^	0.59	373	<10^−6^
HT	*Y. pestis*	723	0.39	723	10^−6^	0.60	723	2×10^−6^
	Multiple	421	0.39	421	<10^−6^	0.60	421	<10^−6^
	All	127	0.38	127	<10^−6^	0.59	127	2.9×10^−5^
	*B. anthracis*	1,109	0.24	853	<10^−6^	0.41	1,109	<10^−6^
	*F. tularensis*	637	0.24	500	<10^−6^	0.41	637	<10^−6^
MC	*Y. pestis*	1,331	0.24	1,153	<10^−6^	0.42	1,331	<10^−6^
	Multiple	733	0.28	596	<10^−6^	0.41	733	<10^−6^
	All	214	0.30	165	<10^−6^	0.40	214	<10^−6^

Summary of GSEA results for protein degree and betweenness centrality of human proteins for three networks: (W) whole human PPI network, (HT) the human PPI network generated by only considering high-throughput experiments, and (C) the human PPI network generated by only considering manually curated PPIs. The “# proteins in group” displays the total number of human proteins with at least one interaction. The “ES” columns display the enrichment score calculated by the GSEA for degree and for centrality. The column titled “# proteins contributing” displays the number of proteins contributing to the ES score.

### Bacterial pathogens target host defense pathways

**Figure 3:**
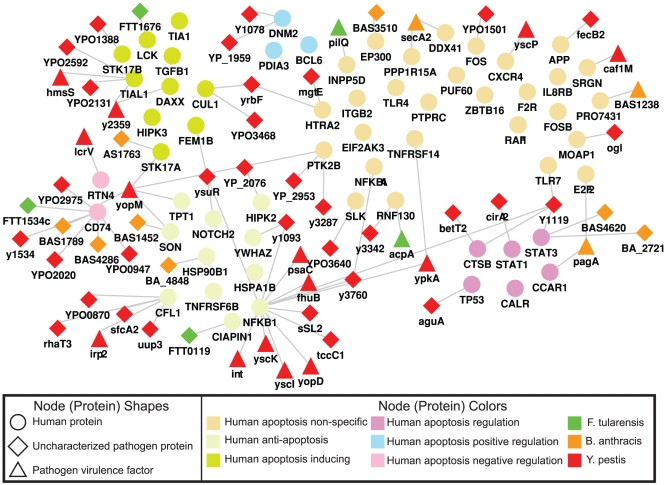
Interactions with host innate immune response. Interactions of human proteins involved in the innate immune response. We divided the human protein into subsets based on whether they induce or prevent apoptosis, or whether they regulate apoptosis. Proteins in the group labeled “Non-specific” do not have an annotation more specific than “Apoptosis” in the Gene Ontology [20]. For clarity this image shows only interactions involving virulence factors and uncharacterized pathogen proteins. As a result, some human proteins in the figure may appear to have no interacting partners.

Since conserved interaction networks between bacterial proteins and the host may be indicative of putative novel targets for broad-based immunotherapeutic development, we asked if human proteins interacting with multiple pathogens may be involved in functions related to host response. Since manipulation of immune responses in the host has been linked to infection by all three pathogens [17], [18], [19], we identified 60 human immune modulation proteins using annotations from the Gene Ontology [20] and their bacterial interactors (see Figure 3). While many of the proteins in the human-respiratory pathogen interaction map play a role in apoptosis, they are also important effectors of immune response signaling. Thus, the double role in apoptosis and immune response regulation should be considered when interpreting these results. This network includes interactions among sets of bacterial and human proteins involved in innate immunity (i.e., TLR4 and TLR7), inflammation (IL-8RB, NF-κB and Bcl-6), recruitment of inflammatory cells, regulatory function, maturation and activation of T cells (i.e., CXCR4, STAT3, NOTCH2, and LCK). For example, LCK is a tyrosine kinase expressed in T cells associated with the cytoplasmic tail of CD4 and CD8 co-receptors. Functionally, LCK is a crucial regulator of T cell activation [21]. Of note, LCK interacts with proteins from all three pathogens, suggesting that these bacteria may have developed conserved mechanisms of impairing effector T cell responses by targeting and possibly disrupting LCK signaling, which is required for inducing acquired immune responses and immune-mediated protection against infectious diseases.

CXC-chemokine receptor 4 (CXCR4) is the major coreceptor for human immunodeficiency virus in CD4^+^ T cells and a promising new target for developing anti-HIV drugs [22]. We find that CXCR4 interacts with the yscP protein, a known virulence factor from *Y. pestis* and a secreted component of the Yop secretion system [23]. The natural ligand for CXCR4 is CXCL12 or SDF1 (stromal cell-derived factor-1) – a chemokine involved in the recruitment of down-modulatory FOXP3^+^ regulatory T cells (Treg) into inflamed tissues [24]. In addition, STAT3 is required for expression of FOXP3 in Treg [25]. Our data demonstrate that STAT3 interacts with the Y1119 protein of *Y. pestis*. In turn, we show that TGF-β1, a down-modulatory cytokine produced by Treg, interacts both with *Y. pestis* and *F. tularensis* proteins. The Schu4 strain of *F. tularensis* has been shown to suppress inflammation in infected mice, and this inhibition has been attributed to induction of TGF-β, another member of the apoptosis PPI network [4]. Similar patterns have been observed in *B. anthracis* and *Y. pestis* [26], [27]. The existence of a putative mechanism of down-regulating immune responses by targeting regulatory pathways merits closer attention.

### Comparative analysis of human-pathogen networks

Encouraged by the evidence in our data suggesting that all three pathogens target proteins involved in host response to infection, we sought to perform a more systematic comparative analysis of the three host-pathogen PPI networks. In preparation for computing conserved modules of host-pathogen PPIs, we used Inparanoid [28] to identify orthologous proteins and OrthoMCL [29] to identify paralogous proteins. From the Inparanoid algorithm we identify a total of 686, 1,179, and 834 pairs of orthologous clusters for the *B. anthracis*-*F. tularensis*, *B. anthracis*-*Y. pestis*, and *F. tularensis*-*Y. pestis* comparisons respectively. We find that 181,505, and 184 of these clusters from the respective comparisons contain more than one protein from either organism. Of these, 93,210, and 129 clusters contain at least one pathogen protein from both organisms that was observed to interact with a human protein in our dataset (see Table 3). We find 1,900 clusters of human proteins from the OrthoMCL analysis.

**Table 3:** Inparanoid ortholog groups.

System	# clusters	# clusters	# clusters
		(>2 proteins)	(pathogen
			interactors)
*B. anthracis*-*F. tularensis*	686	181	834
*B. anthracis*-*Y. pestis*	1,179	505	184
*F. tularensis*-*Y. pestis*	834	210	129

Summary of ortholog groups identified by Inparanoid [28]. The column marked “# clusters (>2 proteins)” is the number of orthologous clusters that contain more than a single protein from each organism. The column marked “# clusters (pathogen interactors)” is the number of orthologous clusters which contain a pathogen protein from each organism that is known to interact with a human protein in our dataset.

First, we performed simple intersections of the detected host-pathogen PPIs. We looked for interologs [30] i.e., a pair of human-bacterial PPIs where the bacterial proteins are orthologous and the human proteins are related. More specifically, we searched for three types of configurations:

- a triple of proteins *a*, *b*, and *c*, where *a* is a human protein that interacts with a protein *b* in one of the three bacteria and with a protein *c* in another bacterium in our data and *b* and *c* are orthologs of each other,
- a quadruple of proteins *a*, *b*, *c*, and *d*, where *a*, *b*, and *c* are as defined before, *d* is a human protein, *a* and *d* interact with each other physically in the human PPI network, *a* interacts with *b* in our data and *d* interacts with *c* in our data, and
- a quadruple of proteins *a*, *b*, *c*, and *d*, where *a*, *b*, and *c* are as defined before, *d* is a human protein, *a* and *d* are paralogs, *a* interacts with *b* in our data and *d* interacts with *c* in our data. As can be seen from Table 4, our interaction data contains a very small number of interologs.

**Table 4:** Conserved interactions.

System	*#ortholog*	*#same*	*#direct*	*#paralogous*
	*pairs*	*protein*	*interaction*	*interactor*
		*pairs*	*pairs*	*pairs*
*B. anthracis* –*F. tularensis*	60	3	0	2
*B. anthracis* –*Y. pestis*	97	5	0	3
*F. tularensis* –*Y. pestis*	98	1	0	3

Summary of bacterial interologs. each row is a pair of bacteria, column 1 is the number orthologous pairs of proteins that both interact with a human protein, column 2 is number of these pairs that interact with the same protein, column 3 is number of these pairs that interact with human proteins that interact themselves, column 4 is number of these pairs that interact with paralogous human proteins.

Since, simple intersections of host-pathogen PPIs did not yield substantial information on conserved PPI networks, we applied four published algorithms for identifying conserved protein interaction modules (CPIMs) amongst the three human-pathogen networks and homology relationships previously identified: Graemlin [31], Match-and-Split [32], NetworkBLAST [33], and GraphHopper [34]. These methods were originally designed to identify conserved modules between intra-species PPI networks. Graemlin requires the user to provide the topology of expected conserved modules as positive examples. Thus, Graemlin was not directly applicable to our scenario since there are no such examples available for these systems. Using the Match-and-Split algorithm we were not able to identify any CPIMs in any of the comparisons. In the case of NetworkBLAST, where there are a number of user parameters that can be adjusted, e.g., complex density and false negative rates, we tried different combinations of values. For the parameter complex density, we varied the input value from 0.50 to 0.95, adjusting values by 0.05 for each test. We performed the same procedure for testing the range of 0 to 0.80 for the FN ratios. Varying the parameters for the NetworkBLAST algorithm had no effect on the identified CPIMs in our case, yielding three CPIMs for the *B. anthracis*-*F. tularensis* comparison, ten CPIMs for the *B. anthracis*-*Y. pestis* comparison, and two CPIMs for the *F. tularensis*-*Y. pestis* comparison. Using the GraphHopper [34] algorithm we were able to identify many more significant CPIMs. In total we identified 39 CPIMs for the *B. anthracis*-*F. tularensis* comparison, 64 for the *B. anthracis*-*Y. pestis* comparison, and 41 for the *F. tularensis*-*Y. pestis* comparison. Table 5 displays the number of identified CPIMs for each of the algorithms. We discuss two sets of CPIMs computed by GraphHooper below related antigen presentation and immune modulation.

**Table 5:** CPIM results.

Alorithm	*B. anthracis*-	*B. anthracis*-	*F. tularensis*-
	*F. tularensis*	*Y. pestis*	*Y. pestis*
Graemlin	N/A	N/A	N/A
Match-and-Split	0	0	0
NetworkBLAST	3	10	2
GraphHopper	39	64	41

Summary of the number of identified CPIMs for each of the algorithms used in this study.

**Figure 4:**
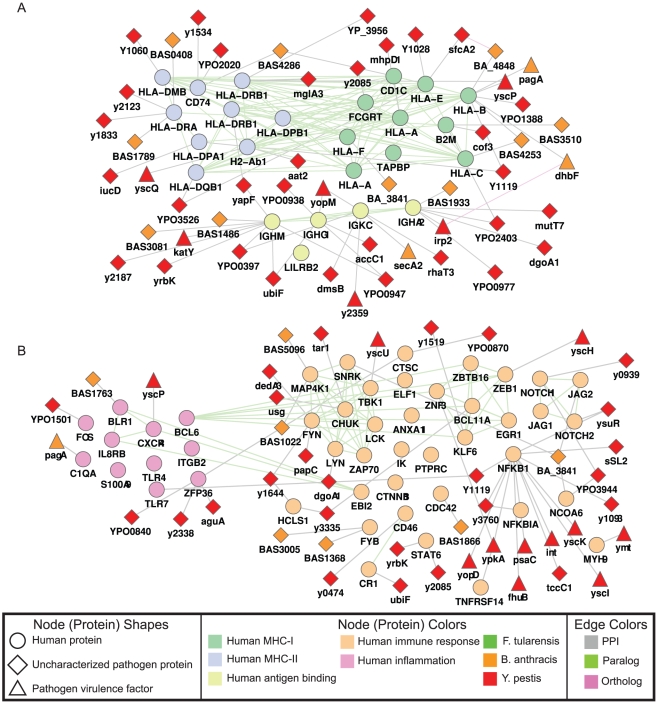
Conserved protein interaction modules. Conserved modules of human-pathogen PPIs involved in (A) antigen binding and processing and (B) immune response pathways. For clarity these images show only the conserved modules from the comparison of *B. anthracis* and *Y. pestis*, and interactions involving virulence factors and uncharacterized pathogen proteins. As a result, the human proteins in the figure may appear to be disconnected.

The major histocompatibility complex (MHC) proteins are responsible for presenting antigens to T cells. Antigen processing and presentation is crucial for activating T cells and mounting protective immune responses. Our analysis captures CPIMs containing human proteins enriched in both antigen processing and presentation functions (Figure 4(a) shows the network for the *B. anthracis* – *Y. pestis* system). We find an interaction between the human HLA-B protein and the *B. anthracis* pagA protein. HLA-B is an MHC class I protein responsible for presenting antigen fragments to CD8^+^ T-cells. The pathogen pagA protein, along with the lethal factor and oedema factor, is one of three proteins composing the anthrax toxin. Functionally, the pagA protein facilitates the translocation of enzymatic toxins across the cell membrane. Also interacting with HLA-B is the *Y. pestis* yscP protein, which is part of the Yersinia outer-membrane protein (YOP) secretion system. Members of the YOP family have been shown to interact with MHC I proteins in the closely related pathogen, *Yersinia enterocolitica* [35]. Other members of the MHC class I family in these CPIMs include HLA-A, HLA-C, and HLA-E. We also identify a number of interactions for human proteins belonging to MHC class II (e.g., HLA-DRA, HLA-DPB1, HLA-DQB1, and HLA-DMB), which are responsible for presenting antigens to CD4^+^ T cells. These MHC class II proteins interact with various pathogen proteins including pathogen membrane proteins and yet uncharacterized proteins.

The CPIMs in Figure 4(b) represent pathogen interactions with human proteins involved in immune response pathways for the *B. anthracis* – *Y. pestis* system. Each CPIM includes NF-κB, which is a transcription factor found at the crossroads of numerous immune and inflammatory pathways leading to the induction of innate and acquired immune responses. NF-κB is found downstream of the Toll family of receptors, which participate in signaling in response to infection. Pathogens have evolved to disrupt this critical process and thereby evade the host response. Inhibition of the NF-κB pathway impairs both the activation and differentiation of T cells and antigen-presenting cells. In the case of *Y. pestis*, the inhibition of the NF-κB pathway is necessary for rapid apoptosis in infected macrophages [36]. We find several members of the *Y. pestis* YOP family, including yscI, yscK, and yopD along with virulence factors such as the toxin tccC1 and the protein kinase ypkA interacting with NF-κB. Many of the other pathogen interactors of NF-κB are labeled as “uncharacterized” proteins. We also observe interactions between the *Y. pestis* proteins usg (an aspartate-semialdehyde dehydro-genase) and tar1 (a methyl-accepting chemotaxis transmembrane protein) and the human IKK-A protein. IKK-A phosphorylates inhibitors of NF-κB, leading to their degradation and resulting in NF-κB activation. We report an interaction between human NFκB-IA, a NF-κB inhibitor that binds to NF-κB and traps it in the cytoplasm, and the *Y. pestis* protein y3760, a putative multi-drug resistance protein. Upstream of NF-κB we demonstrate alr1-TLR4 and Y1119-TLR7 interactions. TLR4 and TLR7 are receptors for LPS and viral single-stranded RNA, respectively. It is well recognized that both *Y. pestis* and *F. tularensis* synthesize LPS with poor TLR4-stimulating activity. However, these further interactions may render NF-κB non-functional. Our findings suggest a strong interaction between bacterial proteins and proteins of the human immune system that are both crucial for effector activity and conserved.

## Materials and Methods

### Experimental Methods

We used a random yeast two-hybrid approach to identify physical interactions between human proteins and pathogen proteins. See Figure 1 for an overview of the experimental analytical processes used in this study.

#### Vectors and strains

The two-hybrid vectors that we used for the random two-hybrid process are based on the *Saccharomyces cerevisiae* Gal4p DNA-binding domain (amino acids 1 to 147 for DBD constructs) and transcriptional activation domain (amino acids 768 to 881 for activation domain libraries). Both vectors have elements suitable for growth in both bacterial and yeast cells. Two DNA binding domain (DBD) cloning strategies were used that differ in the open reading frame (ORF) selection strategy. The DBD fusion vector pOBD.109 has a marker for selection of tryptophan prototrophy (TRP1) and kanamycin resistance. The second DBD is first cloned into the vector pOBD.111 where ORFs are selected using MET2. We then PCR amplified all ORFs and clone them into the fusion vector Super B DBD. The Activation Domain (AD) fusion vector pGAD.PN2 has selection for leucine prototrophy (LEU2) and ampicillin resistance (ampR). In both vectors, expression of the fusion proteins is constitutively driven by the ADH1 promoter. Both vectors also contain centromeric sequences that serve to stably maintain the plasmids and keep the copy number to one or two per cell. For the random two-hybrid experiments, we used a proprietary DNA-binding domain vector that permits the selection of inserts containing open reading frames (pOBD.111). This selection was achieved by inserting a MET2 selectable marker in-frame and downstream of Gal4p DNA-binding domain and the cloning site. In the absence of selection for an in-frame open reading frame (ORF), the majority of inserts will be from non-coding regions or will be out of frame, and therefore of no utility in a two-hybrid assay. Using this ORF selection strategy, greater than 80% of the cloned inserts in these vectors contain open reading frames after nutritional selection. The DNA-binding domain vectors we used, pOBD.111 and pOBD.109, are slightly modified to facilitate the cloning of bacterial genomic DNA fragments that have had linkers added to their ends. The haploid yeast strain used to express the DNA-binding domain fusions, PNY200, has the following genotype: MATα trp1-901 leu2-3,112 ura3-52 his3-200 ade2 gal4 gal80. The haploid yeast strain used to express the activation domain fusions, PJ69-4A1, has the following genotype: MATα trp1-901 leu2-3,112 ura3-52 his3-200 ade2 gal4 gal80 GAL2-ADE2 LYS2::GAL1-HIS3 met2::GAL7-lacZ. The two yeast strains are derived from the same parent cell line and display high mating efficiencies. Both allow for the introduction and selection of vectors carrying the yeast selectable markers TRP1, LEU2, and URA3. The activation domain strain contains three different Gal4p-responsive reporter genes: GAL2-ADE2 and GAL1-HIS3, which are assayed by selection on yeast synthetic media lacking either adenine or histidine, respectively, and GAL7-lacZ, which can be monitored using colorimetric or luminescent assays for beta-galactosidase activity. The HIS3 reporter exhibits a low level of background His3p expression that can be counteracted by use of 3mM 3-amino-1,2,4-triazole, a competitive inhibitor of the His3p protein. These markers are unrelated except for the small GAL4 binding sites in their promoters. Since it is very unlikely that all three genes would be spuriously activated if their promoters are so distinct, the likelihood of false-positives is reduced.

#### Generation of DNA-binding domain libraries

We cloned fragments of *B. anthracis*, *F. tularensis*, and *Y. pestis* genomic DNA into DNA-binding domain vectors pOBD.111 and pOBD.109 to create libraries for two-hybrid analysis. We obtained the genomic DNA from the laboratories of Dr. Kenneth Bradley (University of California, Los Angeles), Dr. Martha Furie (Stony Brook University), and Dr. James Bliska (Stony Brook University) respectively. Bacterial genomic DNA insert preparation involves the mechanical (sonication) and enzymatic (cviJI**) shearing to produce random fragments of an average size of 500 bp. We blunted single-stranded overhangs to recover fragments of desired size. We then ligated purified fragments to linkers and co-transform them into bacterial cells with an equimolar amount of linearized vector. We then transformed the entire ligation and plate onto selection plates for amplification. We pooled colonies and isolated plasmid DNA for transformation into yeast.

#### Preparation of DNA-binding domain fusions

In order to randomly screen each DBD library we plated an aliquot of the DNA-binding domain library on yeast synthetic media lacking tryptophan at a density that allows the selection of individual yeast colonies. After a three to four day incubation, we picked individual yeast clones into a 96 well plate containing yeast rich media (YPD). We then incubated the plate at 30° for one to two days to permit the growth of a sufficient quantity of DNA-binding domain yeast.

#### Random yeast two-hybrid screens

Our strategy is similar to one used by LaCount *et al.* [6] used to identify interactions between proteins in *Plasmodium falciparum*.

We generated DNA-binding domain libraries in a haploid MATα strain and the human spleen activation domain library in a MATα strain. We mated each haploid yeast culture containing a single DNA-binding domain fusion to generate diploid yeast cells that express both the DNA-binding and activation domain fusions. In contrast to LaCount *et al.*, we used a liquid-format mating strategy in a 96-well plate (as opposed to mating on filters or agar), thus allowing for the generation of greater than 500,000 diploids (and, therefore, protein combinations). We selected two-hybrid positives on yeast minimal media lacking tryptophan and leucine (to select for mating events), and lacking histidine and adenine (to select for activation of the two-hybrid reporter genes).

The goal was to analyze the vast majority of *B. anthracis*, *F. tularensis*, and *Y. pestis* proteins as DNA-binding domain fusions. The DNA-binding domain libraries contain fragments sizes selected to be larger than 300 bp and with the average insert size of 500 bp (167 amino acids). We chose the 300 bp minimum because many recognizable protein domains are in this size range; in addition, this size of fragment works well in yeast two-hybrid assays.

We generated comprehensive protein interaction maps by performing a ten-fold coverage of the coding capacity of each of the pathogen genomes. We calculated the number of screens by dividing the total genomic sequence of the pathogen by the average fragment size in the DNA-binding domain library (500 bp) and multiplying by ten (fold coverage).

#### Analysis of positive screens

We incubated the yeast selection plates for ten days. We experienced three different outcomes: 1) Some plates exhibited no growth of yeast colonies and are discarded without further analysis; 2) Some plates exhibited growth of a very large number of colonies (from hundreds of yeast colonies to a lawn of yeast); 3) The remaining plates contained a modest number of colonies, from one to a few hundred. In the first scenario where there are no colonies returned, we assumed that there are no detectable interactors for those protein fragments. In the second case where a very large number of colonies are found, it is likely that the DNA-binding domain fusions possess inherent self-activation ability and were not worthy of further investigation, as they did not represent protein interaction pairs. After analyzing many thousands of searches, it is our experience that DNA-binding domain fusions yielding in excess of 100 colonies per search are likely self-activators. Typically, our ORF-selected DNA-binding domain libraries contain two to five percent self-activating baits, in agreement with the frequencies observed for random fragments of *Escherichia coli* and bacteriophage T7.5 [37], [38].

We selected colonies for further analysis and transfered them to fresh media. We amplified both the DNA-binding and activation domain inserts by PCR and sequenced the resulting products using dye-primer chemistry on capillary instruments. We used the resulting sequence information to identify the interacting protein fragments.

#### Filtering positive interactions

We retained interactions for positive colonies in which the insert is in the correct orientation, contains one but no more than two annotated genes, and does not contain multiple genomic fragments that had been ligated together.

### Computational methods

#### Notation

We represented each experimentally derived human-bacterium protein-protein interaction (PPI) network as a bipartite graph *B* = (*H*, *P*, *I*), where *H* is the set of human proteins, *P* is the set of proteins in the bacterium, and *I* is a set of edges (interactions), each of which connects one protein in *H* to a protein in *P*. Further, we represented the set of known intra-species (human) protein-protein interactions as an undirected graph *G* = (*V*, *E*), where *V* is the set of nodes (human proteins) and *E* is the set of edges (interactions). We now describe in detail the tests we used to analyze each of the human-pathogen networks.

#### Analysis of degree in the human PPI network

The *degree* of a protein in a graph is the number of interactions in which it participates. We plotted the degree distributions for six sets of human proteins: (i) the set of all human proteins not interacting with a pathogen protein in our dataset, (ii)–(iv) three sets of human proteins contained within each of the human-bacteria networks, (v) the set of human proteins found to interact with at least two pathogens, and (vi) the set of human proteins found to interact with all human pathogens (*B. anthracis*, *F. tularensis*, and *Y. pestis*). A bias towards high-degree proteins in the last five distributions would suggest that *B. anthracis*, *F. tularensis*, *Y. pestis* have evolved to interact with higher degree proteins in the human PPI network.

#### Analysis of betweenness centrality in the human PPI network

The degree of a protein captures only its local connectivity. Betweenness centrality (BC) measures capture both global and local features of a protein's importance in a network [39]. A protein with high betweenness centrality is characteristic of a bottleneck in an interaction network (i.e., there are many paths which pass through this protein) [40]. The *betweenness centrality* for a protein *v*∈*V* is defined as the fraction of shortest paths in *G* between all protein pairs (*u*, *w*) that pass through the protein *v*. Given *u*, *v*, *w*∈*V*, let Σ*_uw_* denote the number of shortest paths between proteins *u* and *w*. There may be multiple equally long paths between *u* and *w* that are shorter than any other path between *u* and *w*. Let Σ*_uw_*(*v*) denote the number of these that pass through *v*. Then the betweenness centrality of *v* is
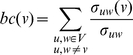
To compute the betweenness centrality for each protein in *G*, we used the algorithm devised by Brandes [41]. This algorithm runs in time proportional to the product of the number of nodes in *G* and the number of edges in *G*. We plotted distributions for the same six sets as in the degree analysis. Again, if the distribution for the last five sets is biased toward higher values of centrality than the distribution for the first set, we could hypothesize that *B. anthracis*, *F. tularensis*, and *Y. pestis* have evolved to interact with proteins with high betweenness centrality in the human PPI network.

#### Gene set enrichment analysis

We used Gene Set Enrichment Analysis (GSEA) to determine if the human proteins interacting with *B. anthracis*, *F. tularensis*, and/or *Y. pestis* have significantly higher degree or betweenness centrality than randomly picked proteins in *G* [16]. Let *L* be the ranked list of the proteins in *V*, where we rank the proteins either by degree or by betweenness centrality. Given *L* and a predefined set *S* of proteins of interest (e.g., those interacting with *B. anthracis*), we used GSEA to determine whether the proteins contained in *S* are randomly distributed throughout *L* or concentrated at the top. In the ranked list *L*, let *l_i_* be the value (of degree or centrality) at index *i*; 1≤*i*≤|*L*|. We abuse notation and say that an index *i* is an element of *S* if the protein whose rank is *i* belongs to *S*. First, we computed *m* = Σ*_i_*_∈*L*_ *l_i_*, the sum of all the values in *L*. Next, for each index *i* in *L*, we computed two values:
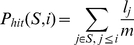


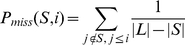
Thus, *P_hit_*(*S*, *i*) measures the weighted fraction of proteins with index at most *i* that are in *S* and *P_miss_*(*S*, *i*) measures the fraction of proteins with index at most *i* that are not in *S*. We handled multiple ranks with identical values by computing *P_hit_* and *P_miss_* only at the largest rank for each unique value in *L*. Finally, we defined the enrichment score as the largest positive value of *P*_hit_(*S*, *i*)−*P*_miss_(*S*, *i*), i.e.,
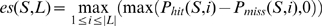
A large positive value of *es*(*S*, *L*) indicates that the proteins in *S* have high degree or high betweenness centrality. Note that our modification of the original definition of the enrichment score [33] ensures that if *S* mainly contains proteins with low degree or betweenness centrality, then the score will be close to 0, since *P*_hit_(*S*, *i*)−*P*_miss_(*S*, *i*) will be negative for most indices. To compute *p*-values for an observed enrichment scores, we generated a null distribution of scores by repeatedly selecting |*S*| random nodes in *L* and computing the enrichment score for each random subset of nodes. We repeated this process 1,000,000 times and estimated the *p*-value for *s* as the fraction of random sets whose enrichment score is at least as large as *s*.

#### Identifying paralogous and orthologous protein pairs

In preparation for computing conserved protein interaction modules, we computed orthologous pairs of proteins in every pair of pathogens. We used Inparanoid [28] with default parameters to define orthologous pairs of proteins. The Inparanoid algorithm outputs pairs of clusters. Each cluster in a pair contains proteins from the same organism. The protein at the center of a cluster has a weight of one and the other proteins in the cluster have a weight between zero and one, depending on their similarity to the protein at the center. In a given pair of clusters, for every pair of proteins (one from each cluster), we use the products of the weights of the two proteins as an estimate of the degree of orthology of the protein pair. In addition, we used OrthoMCL [29] with default parameters and a BLAST e-value cutoff of 10^−10^ to identify paralogous pairs of human proteins. We assigned a weight of one to all paralogous pairs. For the sake of convenience, we considered a human protein appearing in one human-pathogen PPI network to be paralogous to a copy of the same protein appearing in another human-pathogen network.

#### Conserved human-pathogen PPI modules

Given a pair of human-pathogen PPI networks *B*_1_ and *B*_2_, let *Z* be the bipartite graph whose edges are the orthologous and paralogous pairs of proteins between *B*_1_ and *B*_2_, as computed above. We used a weight of one for all edges (the PPIs) in *B*_1_ and *B*_2_. For edges in *Z*, we used the weights defined in the previous sections. Let *w_e_* denote the weight of edges *e* in *Z*. Following the GrapHopper algorithm [34], we defined a *Conserved Protein Interaction Module* (CPIM) to be a triple (*T*_1_, *T*_2_, *O*) where *T*_1_ and *T*_2_ are connected subgraphs of *B*_1_ and *B*_2_, respectively, and *O*⊆*Z* such that (*a*, *b*)∈*O* if and only if *a* is a node in *B*_1_ and *b* is a node in *B*_2_. Thus, *O* is the subgraph of *Z* induced by the nodes in *T*_1_ and *T*_2_. We used two measures of quality for a CPIM: interaction score and conservation score.

We defined the *interaction score* of a CPIM (*T*_1_, *T*_2_, *O*) to be simply the total number of host-pathogen PPIs in *B*_1_ or in *B*_2_ and denoted this score by *q*(*T*_1_, *T*_2_, *O*). Given *T*_1_ and *T*_2_, a small value of the score indicates that we could connect the proteins in *T*_1_ and in *T*_2_ using a small number of PPIs. The conservation score of a CPIM (*T*_1_, *T*_2_, *O*) measures the amount of evolutionary similarity (at the amino acid level) between the human-pathogen interaction networks *T*_1_ and *T*_2_. Let *P*_1_ (respectively, *P*_2_) be the sets of nodes (both human and pathogen) in *T*_1_ (respectively, *T*_2_). We defined the *conservation score* of the CPIM (*T*_1_, *T*_2_, *O*) as
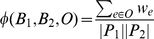
i.e., the total weight of the orthologous or paralogous pairs of nodes in the CPIM divided by the total number of nodes in the CPIM. The larger this score, the more evolutionary conserved we consider *T*_1_ and *T*_2_ to be, since there are fewer proteins without orthologs or paralogs in the CPIM. Note that if we are given *T*_1_ and *T*_2_, we can maximize this score by making *O* the subgraph of *Z* induced by *P*_1_ and *P*_2_.

#### The GraphHopper Algorithm

We used the GraphHopper algorithm [34] to compute CPIMs. For the sake of completeness, we describe the algorithm here. Given two human-pathogen PPI networks *B*_1_ and *B*_2_, GraphHopper finds CPIMs by “hopping” from one network to another using orthology and paralogy relationships. We did not provide PPIs between human proteins as input to GraphHopper. GraphHopper attempts to find CPIMs with high conservation and low interaction score. At a high level, the algorithm starts with a connected basis CPIM that contains four nodes and edges. Iteratively, the algorithm “hops” from one PPI network to another. In each iteration, GraphHopper expands the CPIM to increase the conservation score, while attempting to keep the interaction score as low as possible. We now provide details about the algorithm. Although the GraphHopper algorithm has been described earlier [34], we include these details here in order to make this work self-contained. Our inputs are two human pathogen protein interaction networks *B*_1_ = (*V*_1_, *E*_1_) and *B*_2_ = (*V*_2_, *E*_2_) and a set *Z* of orthologous or paralogous protein pairs.

#### Computing basis CPIMs

We start by constructing a basis set of CPIMs in which each CPIM (*T*_1_, *T*_2_, *O*) has the following properties:

iv. *O* contains two edges (*a*, *a*′)∈*Z* and (*b*, *b*′)∈*Z*;
v. *a* and *b* are connected by at most one intermediate protein in *B*_1_; and
vi. *a*′ and *b*′ are connected by an intermediate protein in *B*_2_.

Thus, each basis CPIM consists of two or four host-pathogen PPIs (one or two each in *T*_1_ and in *T*_2_) and two orthology or paralogy edges. The basis set consists of all such CPIMs.

#### Expanding a basis CPIM

**Figure 5:**
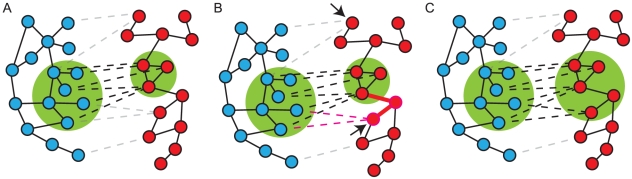
GraphHopper extension of basis CPIM. An illustration of how GraphHopper expands a CPIM in iteration *k*. Each image shown two host pathogen PPI networks, one on the left (blue proteins) and one of the right (red proteins). In these images, we do not distinguish between host and pathogen proteins since GraphHopper treats these equally. Solid edges denote PPIs and dashed edges denote orthologs or paralogs. (A) A CPIM at the end of iteration *k*−1. (B) In iteration *k*, GraphHopper keeps the network in left side of the CPIM fixed and expands the network in the right side of the CPIM. The two nodes marked by arrows belong to the set *P*. The node *v*′ is the lower of these two nodes. GraphHopper adds the thick red interactions and orthology edges to the red network in the CPIM. (C) The CPIM at the end of iteration *k*.

GraphHopper processes each CPIM in the basis set using the following iterative algorithm (see Figure 5). Let (*T*_1_, *T*_2_, *O*) be a basis CPIM. In iteration *k*>1, we construct a CPIM (*T*_1_ *^k^*, *T*_2_ *^k^*, *O^k^*) such that

- (*T*_1_ *^k^* ^−1^, *T*_2_ *^k^* ^−1^, *O^k^* ^−1^) is a subgraph of (*T*_1_ *^k^*, *T*_2_ *^k^*, *O^k^*),
- φ(*T*_1_ *^k^*, *T*_2_ *^k^*, *O^k^*)>φ(*T*_1_ *^k^* ^−1^, *T*_2_ *^k^* ^−1^, *O^k^* ^−1^), i.e., the new CPIM has a higher conservation score, and
- *q*(*T*_1_ *^k^*, *T*_2_ *^k^*, *O^k^*)>*q*(*T*_1_ *^k^* ^−1^, *T*_2_ *^k^* ^−1^, *O^k^* ^−1^) is as small as possible, i.e., the new CPIM has as few PPIs added to it as possible.

We keep either *T*_1_ *^k^* ^−1^ or *T*_2_ *^k^* ^−1^ fixed and “expand” the other graph. Without loss of generality, we assume that *T*_1_ *^k^* = *T*_1_ *^k^* ^−1^ and *T*_2_ *^k^* ^−1^ is a subgraph of *T*_2_ *^k^* in the following discussion. We construct (*T*_1_ *^k^*, *T*_2_ *^k^*, *O^k^*) using the following steps:

- We identify a set *P*⊆*V*_2_ of nodes such that each node *v*∈*P* is not a node in *T*_2_ *^k^* ^−1^ and is connected by an edge in *Z* to at least one node in *T*_1_ *^k^*.
- For each node *v*∈*P*, we use breadth-first search to compute the shortest path π*_v_* in *B*_2_ that connects *v* to *T*_2_ *^k^* ^−1^, i.e., for each node *u*∈*T*_2_ *^k^* ^−1^, we compute the shortest path between *u* and *v* in *B*_2_, and set π*_v_* to be the shortest of these paths.
- We find the node *v*′ in *P* such that π*_v_*_′_ is the shortest among all paths computed in the previous step.
- We set *T*_2_ *^k^* to be the union of *T*_2_ *^k^* ^−1^ and π*_v_*_′_.
- We set *O^k^* to be the union of *O^k^* ^−1^ and the set of edges in *Z* incident on *v*′and a node in *T*_1_ *^k^*.
- We compute φ(*T*_1_ *^k^*, *T*_2_ *^k^*, *O^k^*). If φ(*T*_1_ *^k^*, *T*_2_ *^k^*, *O^k^*)>φ(*T*_1_ *^k^* ^−1^, *T*_2_ *^k^* ^−1^, *O^k^* ^−1^), we go to Step (i) and expand φ(*T*_1_ *^k^*, *T*_2_ *^k^*, *O^k^*) while keeping *T*_2_ *^k^* fixed. Otherwise, we stop expanding this CPIM and proceed to the next basis CPIM.

The rationale for these steps is as follows. To expand the CPIM (*T*_1_ *^k^* ^−1^, *T*_2_ *^k^* ^−1^, *O^k^* ^−1^) after setting *T*_1_ *^k^* = *T*_1_ *^k^* ^−1^, we first identify the set *P* of nodes in *B*_2_ that do not belong to *T*_2_ *^k^* ^−1^ but are orthologs of nodes in *T*_1_ *^k^*. Each node in *P* is a candidate that we can add to *T*_2_ *^k^* ^−1^ in order to construct *T*_2_ *^k^*. However, such a node *v*∈*P* may not be adjacent to any node in *T*_2_ *^k^* ^−1^. Since our goal is to keep *q*(*T*_1_ *^k^*, *T*_2_ *^k^*, *O^k^*)−*q*(*T*_1_ *^k^* ^−1^, *T*_2_ *^k^* ^−1^, *O^k^* ^−1^) as small as possible, we would like to connect *v* to *T*_2_ *^k^* ^−1^ using the fewest edges in *B*_2_. A natural candidate for this set of edges is the shortest path π*_v_* connecting *v* to *T*_2_ *^k^* ^−1^, where this minimum is taken over the set of shortest paths connecting *v* to each node in *T*_2_ *^k^* ^−1^. Therefore, for each node *v* in *P*, we compute the shortest path π*_v_* by which we can connect π*_v_* to *T*_2_ *^k^* ^−1^ using only edges in *B*_2_. We add that path π*_v_* to *T*_2_ *^k^* ^−1^that is shortest among all the paths computed i.e., *v*′ = *arg min_v_*_∈*P*_ |π*_v_*|. After computing *T*_2_ *^k^*, we set *O^k^* to be the subgraph of *Z* induced by the nodes in *T*_1_ *^k^* and *T*_2_ *^k^* by adding the edges in *Z* that are incident on *v*′and any node in *T*_1_ *^k^*; by construction, no node in π*_v_*_′_ other than *v*′ is connected by an edge in *Z* to a node in *T*_1_ *^k^*. This step completes the construction of (*T*_1_ *^k^*, *T*_2_ *^k^*, *O^k^*). Finally, we continue expanding (*T*_1_ *^k^*, *T*_2_ *^k^*, *O^k^*) if its conservation score is greater than φ(*T*_1_ *^k^* ^−1^, *T*_2_ *^k^* ^−1^, *O^k^* ^−1^). Otherwise, we stop the iteration and move on to the next basis CPIM. By induction, the graphs *T*_1_ *^k^*, *T*_2_ *^k^*, and *T*_1_ *^k^* ∪ *T*_2_ *^k^* ∪ *O^k^* are connected. Note that *q*(*T*_1_ *^k^*, *T*_2_ *^k^*, *O^k^*) implicitly plays a role in the expansion: by choosing to add the shortest π*_v_* to *T*_2_ *^k^*, we are attempting to minimize *q*(*T*_1_ *^k^*, *T*_2_ *^k^*, *O^k^*)−*q*(*T*_1_ *^k^* ^−1^, *T*_2_ *^k^* ^−1^, *O^k^* ^−1^).

#### Assessing the statistical significance of a CPIM

We computed the statistical significance of a CPIM using standard methods [33]. We computed two random PPI networks with the same degree distribution as *B*_1_ and *B*_2_ and a random network connecting nodes in *B*_1_ to nodes in *B*_2_ with the same degree distribution as *Z*. We computed a histogram of the conservation scores of all CPIMs that GraphHopper finds in these networks. We amalgamated histograms over 10,000 random inputs and estimated the *p*-value of a CPIM (*T*_1_, *T*_2_, *O*) as the fraction of CPIMs in random networks whose conservation score is at least as large as φ(*T*_1_, *T*_2_, *O*). We retained CPIMs that have *p*-value at most 0.05.

#### CPIM Functional Enrichment

For each CPIM we compute enriched Gene Ontology (GO) [20] functions for five sets of proteins: the set of human proteins interacting with the first pathogen, the set of human proteins interacting with the second pathogen, all human proteins in the CPIM, and each of the two sets of pathogen proteins in the CPIM. For a set of proteins *S*, e.g., those interacting with the first pathogen, we compute enriched functions as follows. For every function *f* in GO, let *s_f_* be the number of proteins in *H* annotated with *f*. Let *u_f_* be the number of proteins in the universe *U* annotated with *f*. As the universe for human proteins, we used the set of all human proteins we have identified in the human activation library (including experiments not described here). For pathogen proteins, we used the set of pathogen proteins found to interact with at least one human protein as the universe. With these counts, we computed the *p*-value of *f* as
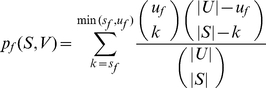
We retained functions only for which *p_f_*≤0.05 after accounting for multiple hypothesis testing using the method of Benjamini and Hochberg [42]. Since functions in GO are specified at multiple levels of detail, the set of enriched function pairs may contain closely related pairs of functions. We used the following criteria to collapse the enriched functions to the most specific and the most enriched. From the set of all enriched functions, we removed a function *f* if there is another function *g* such that

- *p_g_*<*p_f_* i.e., *g* is more statistically significant than *f*, and
- *g* is either an ancestor or a descendant of *f*.

Thus, we retained a function *g* precisely when *g* is more significant than all its ancestors and all its descendants in GO.

#### Merging CPIMs

The steps described above convert each basis CPIM into an expanded CPIM with high conservation and low interaction score. However, the expanded CPIMs may have considerable overlap. We modified the procedure used by Sharan *et al.* [33] to merge CPIMs. For each CPIM *C*, we computed all the biological functions it is enriched in and record the function *f_C_* that is most enriched (has smallest *p*-value) in *C*. Let *F* be the set of all such most-enriched functions. Finally, for each function *l*∈*F*, we computed a CPIM *C_l_* as the union of all CPIMs *C* for which *l* = *f_C_*, i.e., *C_l_* = ∪_l = *Cl*_ *C*. We report results for these CPIMs. Note that this method (i) does not require us to provide a cutoff on the overlap of two CPIMs that should be merged, (ii) allows merged CPIMs to share both proteins and interactions, and (iii) may yield disconnected CPIMs. For each such CPIM, we recomputed the most enriched function. We added other proteins annotated with the function to the CPIM, as long as they participate in a host-pathogen PPI and the pathogen protein is a known virulence factor. Note that the images in the main text only display interactions involving virulence factors and uncharacterized pathogen proteins, for the sake of clarity.

#### Datasets used

We gathered 78,804 PPIs between human proteins from seven databases: the Biomolecular Interaction Network Database [7], the Database of Interacting Proteins [12], the Human Protein Reference Database [11], IntAct [9], the Molecular INTeraction database [13], the Munich Information Center for Protein Sequences [8], and Reactome [10]. For some analyses, we considered a human PPI network assembled from unbiased high-throughput experiments [43], [44], [45] and a network constructed from only manually curated human PPIs [10], [11]. These networks contained 13,172 and 64,427 interactions respectively. We also obtained functional annotations from the Gene Ontology (GO) [20]. We gathered information on virulence factors from MVirDB [46]. These data were downloaded in February 2008.

### Conclusions

In summary, we have provided the first large-scale PPI map for three respiratory bacterial pathogens and their human host. Systematic screening of human-pathogen PPIs also allows us to uncover novel interactions of relevance for understanding pathogenesis, host response, all of which can be applied the development of novel vaccines and immunotherapeutics. In line with recent trends in drug discovery favoring polypharmacology (i.e., drugs acting upon multiple targets), over single target drugs [47], there is a renewed emphasis for developing broadly protective immunotherapeutics against infectious diseases. Accordingly, discovering novel putative targets through the comprehensive lens of protein networks may provide valuable novel insights for developing novel drugs and vaccines against respiratory pathogens.

### Supplementary Information

Information about reagents and the data generated from the yeast two-hybrid screens for *B. anthracis*, *F. tularensis*, and *Y. pestis* are available from the Bioinformatics Resource Center Portal at http://www.pathogenportal.net/prc/. The interactions have also been submitted to the IMEx (http:/www.imexconsortium.org) consortium through IntAct [9] and assigned the identifier IM-13779.
